# Fatal Outcome of Disseminated Strongyloidiasis despite Detectable Plasma and Cerebrospinal Levels of Orally Administered Ivermectin

**DOI:** 10.1155/2009/818296

**Published:** 2009-03-23

**Authors:** Charles E. Rose, Christopher A. Paciullo, David R. Kelly, Mark J. Dougherty, Lawrence L. Fleckenstein

**Affiliations:** ^1^Lexington Infectious Disease Consultants, Lexington, KY 40503, USA; ^2^Department of Pharmacy, University of Kentucky, Lexington, KY 40504, USA; ^3^Commonwealth Neurology, Lexington, KY 40503, USA; ^4^University of Iowa College of Pharmacy, Iowa City, IA 52242, USA

## Abstract

*Strongyloides stercoralis* affects over 100 million people worldwide. Those people most susceptible to infection are those with an immunocompromising condition, such as cancer or human immunodeficiency virus (HIV). Local disease may spread throughout the body of the host, causing a condition termed disseminated strongyloidiasis. Standard treatment for *Strongyloides stercoralis* infection is oral ivermectin. We describe a patient with chronic lymphocytic leukemia diagnosed with disseminated strongyloidiasis two weeks after initial presentation. After repeated dosing of oral ivermectin with no clinical response, serum and cerebral spinal fluid (CSF) concentrations of ivermectin were measured to assess absorption. The peak serum concentration of 49.3 ng/mL correlated with a CSF concentration of 0.14 ng/mL. Despite these concentrations, the patient eventually succumbed to multi-system organ failure. We discuss the reasons for treatment failure and explore the utility of measuring ivermectin concentrations.

*Strongyloides stercoralis* is a common and persistent nematode infecting 100–200 million people worldwide [1]. Endemic regions for *Strongyloides* infection include the Southeastern United States, eastern Europe, Southeastern Asia, Bangladesh, Pakistan, sub-Saharan Africa, the West Indies, and South America [2]. Human infection occurs when infective filariform larvae penetrate through skin which commonly happens in endemic areas when the host bare feet come in direct contact with infested soil. This infection without treatment may persist for up to the lifetime of the host through its ability to endogenously reinfect the host [3]. Most infections in immunocompetent hosts are asymptomatic with the exception of pulmonary and gastrointestinal symptoms during acute and chronic infection's [4]. When the host immunity is lost, *Strongyloides* organisms can migrate through the gastrointestinal (GI) mucosa and enter the blood stream and further disseminate to the skin causing serpiginous and urticarial rashes, the lungs causing edema, patchy and rapidly changing infiltrates, and even causes central nervous system involvement [4, 5]. This dissemination or hyperinfection occurs in patients with human T-cell lymphotrophic virus type-1 infections (HTLV-1), hematologic malignancies, or others receiving corticosteroids, chemotherapy, or immunosuppresives for organ transplantation [3, 4, 6–9].

Oral ivermectin is the treatment of choice for *S. stercoralis* infection in normal [10] and immunocompromised humans [3, 11]. However, patients with severe strongyloidiasis may have unpredictable absorption of oral therapy because of paralytic ileus, functional ileus, or hypoalbuminemia. [3, 7, 8, 12–14]. Unfortunately, no parenteral antihelminthic drugs are licensed in humans, but parenteral ivermectin is commonly used in veterinary medicine. We describe the clinical course of a patient with disseminated stronglyoidiasis who was treated unsuccessfully with oral ivermectin. In an attempt to determine possible causes of treatment failure, ivermectin concentrations in plasma and CSF were analyzed.

## 1. Case Report

A 59-year-old female receiving alemtuzumab for chronic lymphocytic leukemia was admitted to a local hospital near her home in eastern Kentucky in October 2007 for altered mental status of two weeks duration. Admission laboratory values included a white blood count of 5 900/mm^3^, hemoglobin of 10.4 g/dL, platelets of 120 000/mm^3^, but absence of eosinophilia. There was evidence of hypoalbuminemia with serum albumin of 2.4 g/dL. Chest roentgenogram revealed right upper lobe infiltrate, and computerized tomography of the head showed only maxillary and ethmoid sinusitis. However, bloody cerebrospinal fluid (CSF) was found with a nontraumatic lumbar puncture (LP) with 200 000 red cells/mm^3^, 172 white cells/mm^3^, and protein of 750 mg/dL. CSF studies including *Herpes simplex* virus (HSV) polymerase chain reaction (PCR), *cryptococcal neoformans* antigen, and bacterial culture were negative. Despite broad-spectrum antibiotics, the patient encephalopathy and pulmonary infiltrates did not improve, and she was transferred to a tertiary hospital in Lexington, KY, USA.

Neurologic findings at the time of transfer included a Glasgow coma score of five, with no localization to pain. Sputum analysis on admission revealed 1  + white blood cells, gram-positive cocci, budding yeast ,and *Strongyloides stercoralis* was identified in the filariform stage by Gram's stain. Oral ivermectin was started at 15 grams as a single daily dose (~147 mcg/kg) via nasogastric tube [15, 16]. The following day, the ivermectin dose was increased to 30 grams per day (293 mcg/kg) for three weeks to account for the patient large body habitus (102 kg) and questionable ileus [15].

Cerebral angiography did not demonstrate aneurysm or vasospasm. LPs 6 days apart revealed 9 00 and 6688 red cells/mm^3^, respectively, and xanthochromic CSF from the second LP. Protein and white cell numbers were normal both times.

CSF studies, performed as described elsewhere, for *Cytomegalovirus* (CMV) PCR, HSV PCR, Epstein-Barr virus (EBV), JC virus (JCV) PCR, cryptococcal antigen, fungal culture, acid-fast stain and culture, and VDRL were all negative [17]. Stool specimens demonstrated rhabditiform larvae, eggs, and adult *S. stercoralis*, with filariform larvae in the sputum for one week after initiation of ivermectin. The *Stronglyoides* antibody was 0.13 IV (index value) which was negative by laboratory reference values (<1.49 considered negative). Lack of neurologic improvement and persistence of *S. stercoralis* from the sputum led to concern for malabsorption of oral ivermectin. Discussion with the Food and Drug Administration (FDA) for permission to use parenteral veterinary ivermectin led to the measurement of plasma and CSF ivermectin concentrations in collaboration with the University of Iowa by high-performance liquid chromatography [18]. The ivermectin plasma and CSF concentrations were 49.3 ng/mL and 0.14 ng/mL, respectively, on day 10 of oral ivermectin therapy. Repeat microscopy of sputum after three weeks of therapy did not reveal organisms. Despite 22 days of oral therapy, the patient showed no sign of neurologic recovery. After discussion and permission from the surrogate decision maker, respiratory support was withdrawn and the patient expired. The family refused autopsy.

## 2. Discussion

This report describes an immunocompromised patient with disseminated *Strongyloides* presenting to a community hospital with two weeks of altered mental status. On transfer to a tertiary hospital, repeat LP confirmed evidence of hemorrhagic CSF with persistent elevation in CSF red blood cells and xanthrochromia. *S. stercoralis* organisms were observed on sputum Gram stain, which was subsequently confirmed in stool and additional sputum samples. Despite immediate institution of ivermectin after transfer to the tertiary care center, the patient did not improve neurologically and expired after being transitioned to comfort care.

The patient may have died from subarachnoid hemorrhage and advanced encephalitis secondary to disseminated strongyloidiasis. A previous report of *S. stercoralis* showed an erythrocytic CSF predominance [14]. The hemorrhagic CSF and xanthochromia were likely secondary to capillary rupture or local inflammation from the host immune response. Previous neuropathology has demonstrated the presence of the *Strongyloides* larvae within capillaries, meninges, and brain tissue in a case of disseminated strongyloidiasis after administration of corticosteroids [19].

As in this patient, amajor difficulty in disseminated strongyloidiasis is overcoming the delay in diagnosis both because of delayed presentation for medical evaluation and the vague symptoms that accompany disseminated disease. However, pulmonary and gastrointestinal abnormalities with an infiltrate and ileus in an immunocompromised host should warrant consideration of the diagnosis [20]. Eosinophilia may be mild and nonspecific and because of low parasite load and irregular larval output. Microscopy of a single stool specimen not surprisingly fails to detect larvae up to 70% of the time [5]. *Strongyloides* antibody may show cross-reactivity with other helminth infections including filariasis, *Ascaris lumbricoides*, and acute schistosomiasis [21, 22]. While serology has a negative predictive value of 95%, our patient had a negative antibody test for *Strongyloides* despite microscopic evidence of pulmonary and gastrointestinal diseases [23]. It is possible that false-negative serology in our patient was related to immunosuppression from either her hematologic malignancy or chemotherapy.

While delay in diagnosis may have contributed to the poor outcome, inadequate central nervous system penetration of ivermectin or ivermectin neurotoxicity may have played a role. Bowel obstruction and ileus are commonly described in hyperinfection syndrome, and intestinal inflammation, and bowel edema from penetration of *Strongyloides* may impair absorption. In this situation the absorption of oral medications is unclear, and determination of ivermectin concentrations appears useful to assess adequacy of oral treatment. Hypoalbuminemia may also affect the pharmacokinetics and distribution of ivermectin. Ivermectin is highly bound to serum albumin, and lower levels of albumin can increase clearance of unbound drug as well as additional effects of contribution to tissue edema which can slow absorption and increase the volume of distribution [14]. Increased clearance or volume of distribution may interfere with achievement of effective levels in the central nervous system.

We were able to achieve a relatively high ivermectin plasma level in our patient, with a daily dose of 293 *μ*g/kg. The plasma concentrations in our patient were similar to those observed in healthy volunteers following multiple oral doses of 60 mg given on days 1, 4, and 7 [24]. Because the concentrations were similar to those observed in healthy volunteers after multiple oral doses, we concluded that there was adequate intestinal absorption and we did not further pursue use of parenteral ivermectin [24].

Despite the higher oral ivermectin dose and achievement of plasma concentrations that were higher than circulating ivermectin plasma levels in previous patients, our patient did poorly [14, 25]. Unfortunately, the effective therapeutic plasma concentration in humans is not known. Therefore, establishing CNS penetration by measuring adequate CSF concentrations may be important in the prediction of outcome. At steadystate, the CSF concentration in our patient suggested less than 1% CSF penetration of orally administered ivermectin. Moreover, in the previous study by Turner et al. after five daily subcutaneous ivermectin doses of 200 *μ*g/kg, there were no detectable levels in the CSF using the same assay methodology used in this study [25]. The plasma levels measured after the initial dose and the seventh daily dose were 5.8 ng/mL and 12.1 ng/mL, respectively, and at 14 days steady-state plasma concentrations were 11.4–17.4 ng/mL [25].

Lastly, it seems unlikely that neurotoxic side-effects of ivermectin contributed to the poor outcome of the patient. Adverse neurologic effects include mydriasis, ataxia, tremors, emesis, lethargy, and coma [13, 24–26]. However, our patient was comatose prior to and after three weeks of therapy. Moreover, healthy volunteers have tolerated single doses of 2 000 *μ*g/kg, and doses up to 1 091 *μ*g/kg given three times at 72-hour intervals without evidence of toxicity [24].

When orally administered ivermectin therapy fails to produce a clinical response, alternate methods of therapy should be considered. Subcutaneous ivermectin has been used in numerous cases to overcome poor oral absorption [9, 14, 25, 27, 28]. While no parenteral antihelminthic medications are FDA approved for use in humans, permission may be obtained on an individual basis to administer these agents by alternative routes with evidence of problems of enteral absorption.

## 3. Conclusion

The mortality in disseminated strongyloidiasis is high, likely from progression of disease prior to diagnosis. Ivermectin remains the drug of choice and is typically given orally at a dose of 200 *μ*g/kg per day for two weeks or until resolution of symptoms. Because of possible impaired absorption, hypoalbuminemia, or extensive central nervous system disease, serum concentrations may help discriminate when either higher oral doses or parenteral ivermectin is necessary. Two case reports as well as ours showed microscopic evidence of *Strongyloides* eradication with serum concentrations ranging from 11.4 to 49.6 (ng/dL). We were able to measure a CSF ivermectin concentration of 0.14 ng/dL, which suggests a less than 1% central nervous system penetrance. Because of extensive mortality, early diagnosis and treatment are paramount in disseminated strongyloidiasis.
